# Factors Associated with Transport Instability and Outcome Deterioration During Interhospital Intensive Care Transfers of Patients with COVID-19 in Germany

**DOI:** 10.3390/jcm15176747

**Published:** 2026-08-31

**Authors:** Leonie Hannappel, Jan Wnent, Rolf Lefering, Janina Bathe, Jan-Thorsten Gräsner

**Affiliations:** 1 Institute for Emergency Medicine, University Hospital Schleswig-Holstein, 24105 Kiel, Germany; jan.wnent@uksh.de (J.W.); janina.bathe@uksh.de (J.B.); jan-thorsten.graesner@uksh.de (J.-T.G.); 2 Department of Anaesthesiology and Intensive Care Medicine, Campus Kiel, University Hospital Schleswig-Holstein, 24105 Kiel, Germany; 3 Institute for Trauma Research of AUC, Academy for Trauma Surgery, 80538 Munich, Germany

**Keywords:** COVID-19, SARS-CoV-2, critical care, patient transfer, transportation of patients, risk factors, time factors, treatment outcome, emergency medical services

## Abstract

**Background/Objectives:** The COVID-19 pandemic created demand for interhospital transfers of critically ill patients to balance intensive care capacity. Evidence on the logistics and immediate physiological outcomes of such transfers in Germany remains limited. **Methods:** This multicenter retrospective observational cohort study included 414 interhospital intensive care transport records of adults with COVID-19 in Germany between January 2020 and June 2021. Logistic regression assessed factors associated with transport instability and deterioration, and linear regression assessed factors associated with transfer-phase duration. **Results:** Transport modality was incompletely documented; 404 records documented ground-based intensive care transport by intensive care transport vehicle (ITW) and 10 by fixed-wing transport. Respiratory support was frequently documented, with intubation recorded in 71.3% of transports, catecholamines in 64.1%, and ECMO transport in 7.2%. Mean takeover, transport, and handover times were 53, 59, and 64 min, respectively. At least one clinically relevant threshold-crossing event during transport was documented in 43.9% of records, while 15.9% met the study-specific definition of transport instability requiring at least two separate threshold-crossing events. Outcome deterioration occurred in 16.4%. In the multivariable analyses, serious impairment of physiology was associated with transport instability, while instability during transport was strongly associated with deterioration at handover. **Conclusions:** Physiological instability and immediate deterioration were frequent in this selected multicenter cohort. Because documentation was heterogeneous, providers participated voluntarily, and longer-term outcomes were unavailable, the findings should not be interpreted as establishing transport-related causation or overall transport safety.

## 1. Introduction

The COVID-19 pandemic posed an unprecedented challenge to healthcare systems worldwide. The rapid spread of SARS-CoV-2 led to a surge in critically ill patients requiring advanced respiratory support and intensive care. Early reports from Italy and France highlighted the overwhelming burden on regional hospitals, with capacity shortages forcing patient transfers across regions and even national borders [1]. These developments underscored the importance of safe interhospital transport for critically ill patients during the pandemic.

In Germany, healthcare provision is highly decentralized, with intensive care transport services regulated at the state level and delivered by a mix of public and private providers, including fire brigades, aid organizations, and aeromedical services. Despite Germany’s high intensive care capacity, its uneven regional resource distribution necessitated large-scale COVID-19 patient transfers during infection peaks.

Before COVID-19, the literature on intensive care transfers focused on intrahospital transport complications [2], air transport safety [3], or logistics for acute respiratory distress syndrome (ARDS) patients [4]. Complication rates in critically ill patient transfers varied widely, with some studies reporting up to 38% deterioration in outcomes [5] and others suggesting that, under structured conditions, interhospital transfers were safe and did not increase mortality [6,7]. However, comprehensive data on large-scale, pandemic-related interhospital transports were lacking at the outset of COVID-19. International studies showed that large-scale interhospital transfers were feasible but logistically challenging [8,9,10].

For Germany, however, systematic analyses of COVID-19 patient transfers were absent at the beginning of the pandemic. The “cloverleaf” system introduced in late 2020 established regional coordination hubs to facilitate cross-state redistribution of patients, but its effectiveness and clinical impact had not been empirically assessed. Particularly vulnerable were patients requiring invasive ventilation, extracorporeal membrane oxygenation (ECMO), or vasopressor support—interventions associated with high risks during transport. Moreover, there was no unified national documentation system for intensive care transfers despite earlier recommendations by the German Interdisciplinary Association for Intensive and Emergency Medicine (DIVI) [11]. The absence of standardized data has limited opportunities to evaluate outcomes, quality, and risk factors.

Against this backdrop, the present study was designed as a multicentre investigation of interhospital transfers of critically ill COVID-19 patients treated by selected transport providers in Germany. It aimed to describe transport characteristics, patient status at takeover and handover, complication rates, and patient- and system-related factors associated with outcomes and transfer duration. Given the voluntary provider participation and substantial regional clustering, the cohort was not intended to be nationally representative.

**Figure 1:**
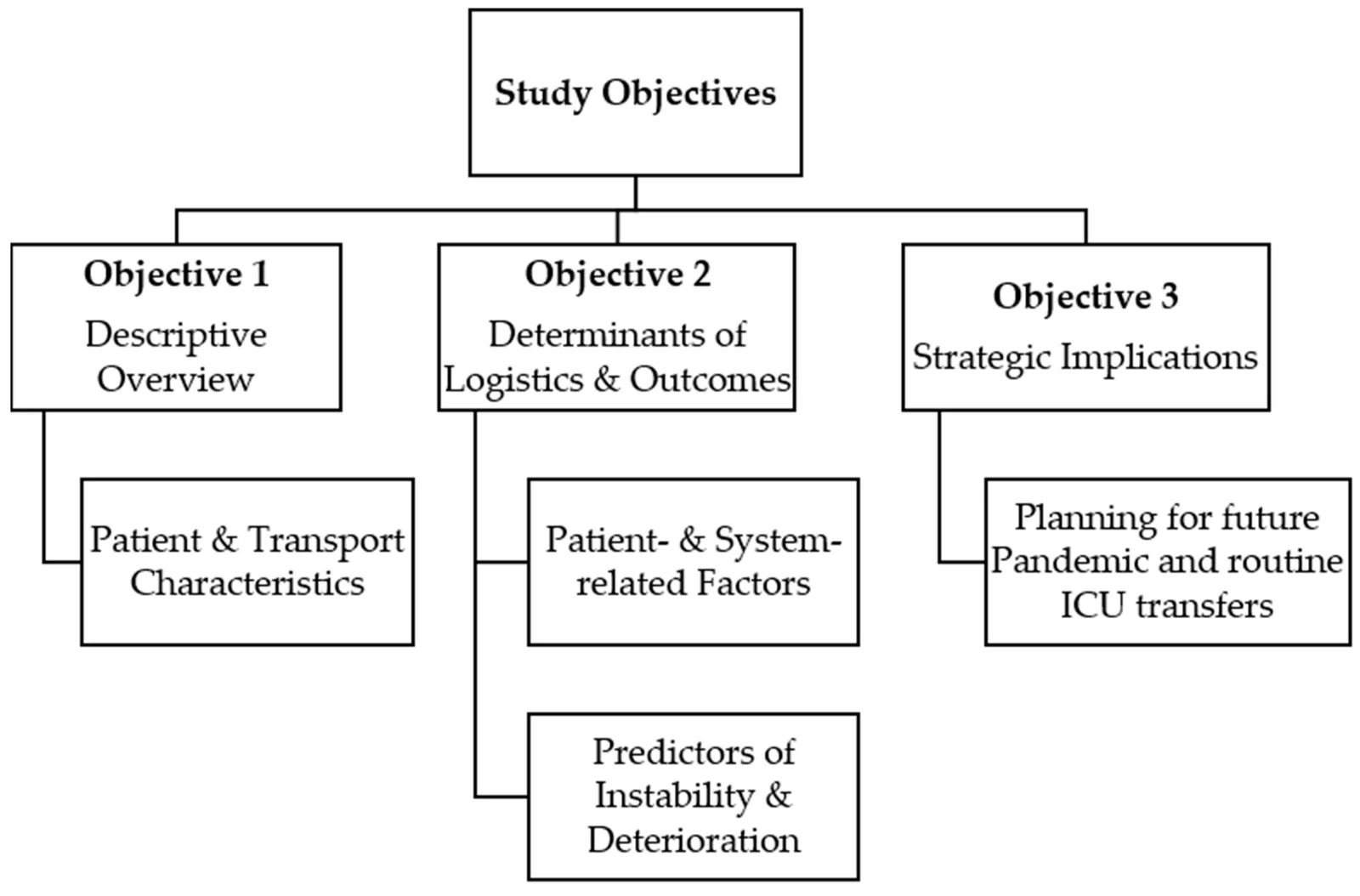
Study Objectives.

The study aimed to identify factors associated with transport instability, deterioration, and transfer logistics (Figure 1).

The study improves our understanding of intensive care transport under pandemic conditions and provides evidence for quality assurance and future preparedness. The findings are of direct public health relevance, as they highlight systemic challenges and risks in the interhospital transfer of critically ill patients, informing preparedness, resource allocation, and coordination strategies during large-scale health emergencies such as pandemics.

## 2. Materials and Methods

### 2.1. Study Design

This investigation was conducted as a multicentre, retrospective observational cohort study within health services research. Baseline clinical and operational characteristics documented at takeover were related to events and physiological changes documented during transport and at handover. The primary transport records were analysed retrospectively; supplementary organizational data were collected prospectively from participating providers.

### 2.2. Patient Population

The study included adult patients with confirmed COVID-19 who underwent intensive care transport between January 2020 and June 2021. A total of 414 transport records were obtained and analysed. Because the dataset did not contain a patient identifier that permitted reliable linkage across transport records, it was not possible to determine whether individual patients underwent more than one transfer. All known intensive care transport vehicle (ITW) locations in Germany were approached repeatedly and invited to submit anonymized copies or scans of transport reports for eligible COVID-19 intensive care transports performed during the study period; participation and submission of records were voluntary. Providers were asked to submit the available documentation for all eligible transports rather than selected individual cases. Records were ultimately received from six ground-based ITW providers (Augsburg, Regensburg, Warendorf, Kiel, Hamburg, Cambs) and one fixed-wing aeromedical provider in Cologne. Augsburg and Regensburg supplied the two largest series and were considered representative of their local ITW activity. Data on helicopter transports were not available. Participation was voluntary. The source material does not provide a national denominator for all COVID-19 intensive care transports during the study period, so a population-level response rate or national coverage proportion cannot be calculated. The completeness of documentation was lower than intended, despite requesting routine reports for all eligible transports. Similar limitations in routine documentation may plausibly have affected transports elsewhere in Germany, but this cannot be demonstrated from the present dataset.

### 2.3. Data Collection

The primary data source was the DIVI intensive care transport report, where available (see translation in the Supplementary Materials). Alternative provider-specific routine forms were largely identical or overlapping with the DIVI form for the core variables used in this analysis, with mainly local modifications or additional fields. A predefined data dictionary was used to map all formats to common variables. Data were digitized, standardized, plausibility-checked, and coded; physiologically implausible values were treated as not documented, and free-text entries were assigned to predefined categories. The routine transport reports did not consistently contain a dedicated field for the reason for transfer. A supplementary provider questionnaire therefore captured transfer reasons, distance, coordination structures, and additional resource requirements.

### 2.4. Definitions and Variables

Three composite variables were defined:

- **Serious impairment of physiology** at transport start, defined by the presence of one or more of the following: catecholamine therapy, fraction of inspired oxygen (FiO_2_) ≥ 0.5, ratio of arterial oxygen partial pressure to fraction of inspired oxygen (P/F ratio) < 100 mmHg, mean arterial pressure (MAP) ≤ 60 mmHg, or positive end-expiratory pressure (PEEP) > 12 cmH_2_O.
- **Instability during transport**, defined as at least two clinically relevant threshold-crossing events in heart rate (<50 bpm or >100 bpm), systolic blood pressure (<90 mmHg or >160 mmHg), oxygen saturation (<90%), or end-tidal CO_2_ (<33 mmHg or >43 mmHg). Events could involve the same or different parameters but had to occur as separate events during transport. Pre-existing abnormalities were not counted unless a corresponding threshold-crossing event occurred during transport.
- **Deterioration after transport**, defined as a decrease in MAP of ≥20%, decline in the Glasgow Coma Scale of ≥2 points, decline in oxygen desaturation of ≥4%, or initiation of catecholamines during transport. For the neurological component, a decline in GCS was classified as deterioration only when it represented an unplanned change in neurological status. Changes attributable to planned initiation or modification of sedation, analgesia, or other intended treatment adjustments related to transport were not classified as deterioration

The composite outcomes were study-specific operational definitions and have not been externally validated as composite endpoints. The underlying physiological thresholds were informed by pre-existing expert consensus developed within the interdisciplinary COVRIIN group during the COVID-19 pandemic [12]. For the present study, these criteria were adapted to the variables available in routine transport documentation and operationalized in consultation with experienced clinicians in Critical Care transport. Their combination into the composite outcomes used here is therefore study-specific and should not be interpreted as a validated clinical score.

### 2.5. Outcome

The primary outcome was patient instability during transport and deterioration after transfer. Secondary outcomes included takeover time at the sending hospital, duration of transportation, handover time at the receiving hospital, frequency of necessary interventions during transport, monitoring adequacy, and factors influencing delays.

### 2.6. Statistical Analysis

Data were entered into Statistical Package for the Social Sciences (SPSS, version 29, IBM Inc., Armonk, NY, USA). Continuous variables were checked for plausibility and summarized as mean with standard deviation or median with quartiles and range, depending on distribution; categorical variables were reported as frequencies and percentages. Multiple linear regression was used for takeover and handover times, and binary logistic regression was used for instability and deterioration. For binary predictors used in the regression analyses, undocumented items were coded as “no” since routine documentation frequently covers just the performed interventions while non-performed interventions were not documented as such. Candidate factors for the two main outcomes were first examined individually. Variables showing a potentially relevant signal were then discussed with an expert group regarding clinical plausibility and suitability for the multivariable model. For logistic regression, the number of predictors was limited using the rule of approximately one predictor per ten outcome events to reduce overfitting. Formal multicollinearity diagnostics were not performed; instead, the expert selection aimed to include parameters from different clinical domains and to avoid obviously strongly correlated predictors. Nagelkerke’s r^2^ was given for each logistic model, and the area under the receiver operating characteristic curve with 95% confidence interval (CI) was used as a measure for discrimination. Formal calibration analyses of the logistic models and additional formal diagnostic assessment of the assumptions of the linear regression models were not performed. The regression analyses were exploratory and were not intended to develop or validate a clinical prediction model.

### 2.7. Ethical Considerations

The study was reviewed and approved by the Ethics Committee of the Medical Faculty of Christian-Albrechts-University of Kiel (protocol D 573/20; positive decision on 1 September 2020). The ethics approval covered the study at all participating sites; no additional local ethics approvals were required. The study used anonymized or pseudonymized routine transport documentation; no direct access to providers’ original identifiable records was available to the study team.

## 3. Results

### 3.1. Operational and Tactical Data

The standardized DIVI form was used in 191/414 records (46.1%), while 223/414 (53.9%) used alternative provider-specific routine documentation. Transport modality was incompletely documented. Of the full cohort, 404 records documented transport by ITW and 10 documented fixed-wing aeromedical transport; no helicopter transport records were available. Provider contributions were strongly clustered: Augsburg contributed 178 records (43.0%) and Regensburg contributed 158 (38.2%), together accounting for 81.2% of the cohort; Warendorf contributed 25 (6.0%), Hamburg contributed 22 (5.3%), Kiel contributed 16 (3.9%), Cambs contributed 2 (0.5%), and Cologne contributed 10 (2.4%); provider location was missing in 3 records (0.7%). Augsburg and Regensburg provided the two largest local series and were considered representative of their respective ITW activity; the overall multicenter cohort, however, was not intended to be nationally representative.

Physician staffing was dominated by anaesthesiologists (98% of reported cases). Where qualification was specified, 80% were specialists and 20% were residents in training.

**Figure 2:**
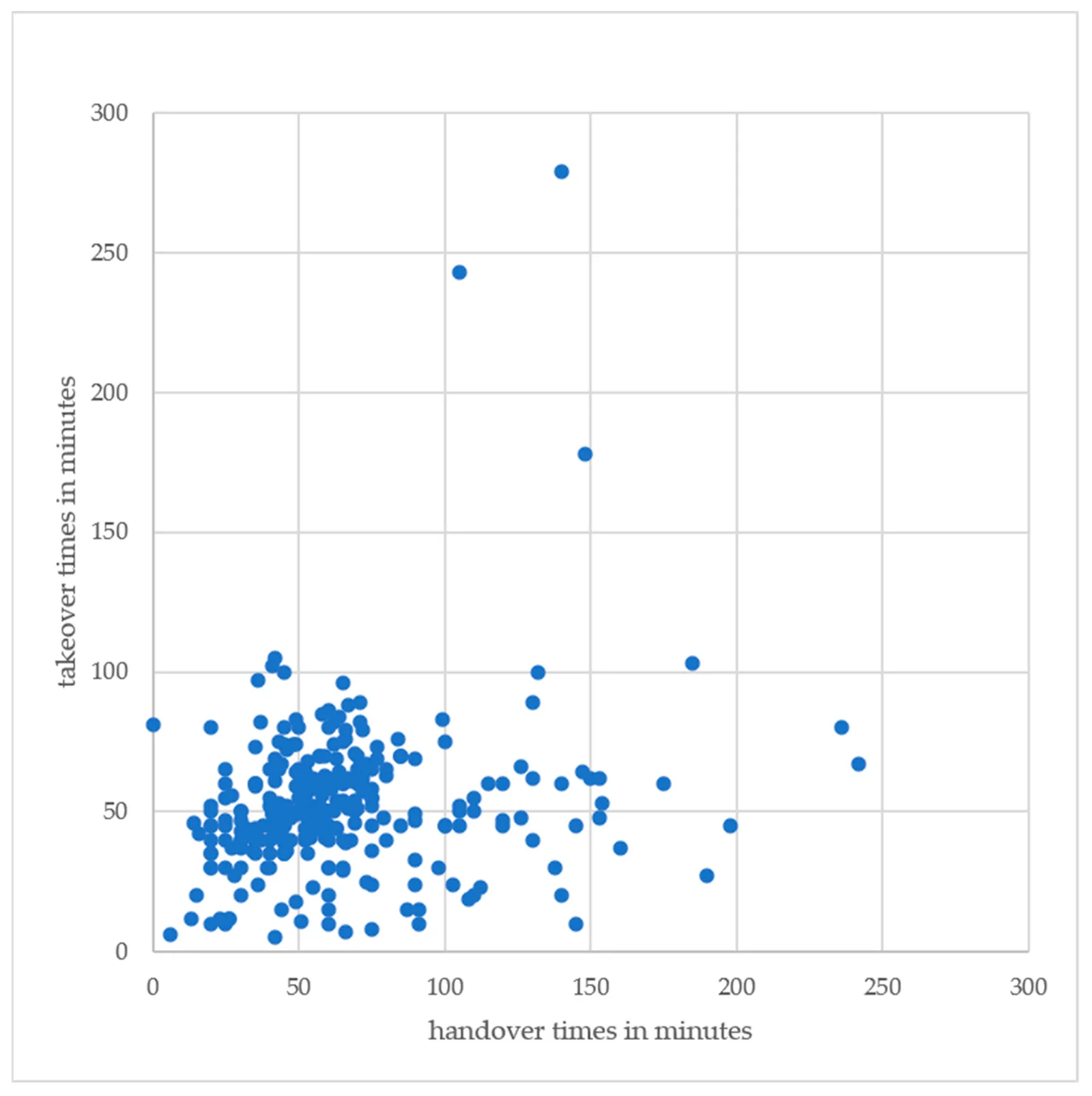
Takeover times compared to handover times for ground-transported patients with available timing data (Pearson’s r = 0.223).

Transfer timing varied widely. Patient takeover averaged 53 min (n = 360; range: 4–279), pure transport time averaged 59 min (n = 369; range: 6–415), and handover at the receiving facility averaged 64 min (n = 305; range: 0–241) (Figure 2).

Linear regression analysis indicated that high priority (as stated in the patient care report) was associated with a ~10-min shorter takeover time, while unconsciousness/sedation (+7 min), catecholamine therapy (+9 min), obesity (+14 min), and ECMO support (+21 min) were associated with longer takeover times (Table 1).

For handover, catecholamine therapy (+11 min) and ECMO (+20 min) were significantly associated with longer handover times. No significant associations were found for hypertension (as comorbidity), age, sex or physician seniority (Table 2).

Most transfers (94%) occurred during pandemic “wave” phases as defined by the Robert Koch Institute, with the majority in the second wave (51%) and third wave (28%).

### 3.2. Priority for Transfer

Priority was documented in 277/414 cases (66.9%): 133/277 (48.0%) were planned within 24 h, 110/277 (39.7%) were urgent within two hours, 30/277 (10.8%) were immediate within 30 min, and 4/277 (1.4%) were scheduled for subsequent days. The routine transport report did not provide a consistently completed field for transfer indication; reasons were instead obtained from the supplementary questionnaire. In the supplementary questionnaire, 36 entries (9%) were not documented, 187 (45%) indicated capacity or resource limitations at the referring hospital, 161 (39%) indicated worsening clinical status or a need for escalation/upward transfer, and 30 (7%) indicated improving clinical status or step-down/downward transfer. The capacity category is consistent with the pandemic context, in which availability of staffed intensive care and ventilator beds was a major operational constraint. These data were used descriptively rather than for reliable subgroup modeling.

### 3.3. Patient Demographics

Sex was missing in 137/414 records (33.1%); among 277 records with sex documented, 186 (67.1%) were male and 91 (32.9%) were female. Age was missing in 33/414 records (8.0%); among the 381 with age data, the largest groups were 60–69 years (107; 28.1%), 50–59 years (87; 22.8%), and 70–79 years (84; 22.0%).

### 3.4. Patient Status at Takeover

Neurological status was assessed using both the Glasgow Coma Scale (GCS) and the four-level consciousness classification recorded in the transport documentation (oriented, clouded, unconscious, or sedated). Although GCS was available in 293/414 records (70.8%), a consciousness-category entry was available in 375/414 (90.6%), and at least one of these two neurological descriptors was available for nearly all transports. The categorical assessment therefore complemented missing GCS entries for the descriptive characterization of consciousness but was not used to impute a numerical GCS value. Most documented patients were sedated (284/375; 75.7%); 71/375 (18.9%) were oriented. Among records with GCS data, 176/293 (60.1%) had GCS 3 and 72/293 (24.6%) had GCS 15.

At takeover, serious impairment of physiology was present in 320/414 patients (77.3%; 95% CI: 73.3–81.3%). Regarding the predefined predictor variables reflecting patient status and treatment at takeover, obesity was present in 32/414 patients (7.7%), 295/414 (71.3%) were intubated, 283/414 (68.4%) received analgesics, and 173/414 (41.8%) received volume therapy.

Cardiovascular variables were more complete: systolic blood pressure was available in 372/414 records (89.9%; median: 123 mmHg), MAP was available in 352/414 (85.0%; median: 81 mmHg), and heart rate was available in 375/414 (90.6%; median: 82/min). Catecholamine dependency was documented in 227/354 records with circulatory status (64.1%). Respiratory documentation did not permit ventilation mode labels to be equated reliably with invasive ventilation. Intubation was documented in 295/414 records (71.3%), while at least one ventilation-mode entry was present in 310/414 (74.9%); recorded modes included bilevel positive airway pressure (BiPAP), continuous positive airway pressure (CPAP), pressure-controlled ventilation (PCV), assisted spontaneous breathing (ASB), controlled mechanical ventilation (CMV), and other modes, with multiple mode entries possible. FiO_2_ was available in 317/414 (76.6%; median: 0.6), PEEP was available in 321/414 (77.5%; median: 12 cm H_2_O), oxygen saturation was available in 357/414 (86.2%; median: 94%), and P/F ratio was available in 288/414 (69.6%; median: 155 mmHg).

### 3.5. Measures and Monitoring

Patients frequently required invasive lines: peripheral intravenous catheters, central venous catheters, and arterial lines were common. ECMO transport occurred in a small subgroup and was associated with longer takeover and handover times. The study was not designed specifically around maximally unstable or ECMO-dependent patients; rather, it examined interhospital transfer of isolation-requiring, frequently ventilated COVID-19 patients during a period of constrained intensive care resources.

Medication and monitoring fields were variably complete. For example, electrocardiographic monitoring was documented in 387/414 records (93.4%), pulse oximetry in 410/414 (99.0%), capnometry in 296/414 (71.5%), and invasive blood pressure monitoring in 241/414 (58.2%). ECMO transport was documented in 30/414 records (7.2%). Missingness was especially pronounced in some handover variables: GCS at handover was unavailable in 136/414 records (32.9%), circulatory status in 137/414 (33.1%), and ventilation information in 136/414 (32.9%).

### 3.6. Instability During Transport

At least one clinically relevant threshold-crossing event during transport was documented in 182/414 records (43.9%). Of the full cohort, 66/414 (15.9%; 95% CI, 12.2–19.5%) met the study-specific definition of transport instability, requiring at least two separate threshold-crossing events during transport. Among 320 patients meeting the study-specific definition of serious impairment of physiology, 162 (50.6%) had at least one documented threshold-crossing event during transport, compared with 20/94 among those not meeting that definition.

The following predictors were considered as independent predictors for instability during transport in a logistic regression analysis: serious impairment of physiology, analgesics, crystalloids/colloids, obesity, and intubation. The first three predictors were found to be statistically significant (Table 3). Nagelkerke’s r^2^ was 0.106, and the area under the receiver operating characteristic curve (AUROC) was 0.69 (95% CI: 0.63–0.75).

### 3.7. Deterioration After Transport

A deterioration after the transfer during handover at the receiving hospital occurred in 68 patients (16.4%, 95% CI: 12.9–20.0%). The multivariable logistic regression analysis considered the same predictors as above, plus an observed instability during the transport (observed in 66 cases, 16.4%), and a rather long transportation time of two hours or more (observed in 20 cases, 4.8%). About half of patients with a deterioration at handover had experienced a transport instability (31/68), and half of those with an instability during transportation had a deterioration at handover (31/66).

Instability during transport was the most predictive factor for deterioration, followed by a serious impairment of physiology (Table 4). Nagelkerke’s r^2^ was 0.209, and the AUROC was 0.75 (95% CI: 0.69–0.82).

### 3.8. Summary of Key Results

- Takeover and handover times were prolonged by patient complexity, particularly ECMO and catecholamine dependency.
- The majority of patients were critically ill, frequently requiring respiratory support and vasopressors.
- Instability during transport and deterioration at handover occurred in about one in six cases.
- In the multivariable analyses, serious impairment of physiology was associated with transport instability, while instability during transport showed the strongest association with deterioration at handover.

## 4. Discussion

### 4.1. Immediate Post-Transport Deterioration

A central finding is that 68/414 transport records (16.4%) met the study-specific definition of immediate deterioration and that physiological instability was frequently documented during transport. These observations identify associations that may inform future risk assessment, but the study cannot determine whether transport caused the observed changes. Previous studies generally found no increase in mortality associated with interhospital transfer, although transient physiological deterioration has been reported [6,7,10,13,14,15].

Other investigations documented substantially higher rates of deterioration. Differences in reported deterioration rates across studies are likely explained by heterogeneity in definitions, transport modalities (e.g., ground vs. air), patient selection, and the extraordinary operational conditions during the COVID-19 pandemic. In one study, 38% of patients worsened after transfer [5], while evacuations from Mayotte to Réunion showed deterioration of the P/F ratio in 34% of cases, particularly in patients with hypertension or diabetes [13]. In France, 82% of critically ill patients transported by air had worsening oxygenation, which correlated with transport duration [13]. It should be noted that P/F ratio values were sometimes estimated from peripheral oxygen saturation (SpO_2_)/FiO_2_ rather than PaO_2_/FiO_2_ [13,14,15]. Additional research linked P/F ratio < 100 mmHg and hemodynamic instability to higher three-month mortality after transfer [13].

Signs of deterioration were reported immediately after transport in 16.4% of patients, despite previous reports describing interhospital intensive care transport as generally safe under structured conditions [6,7]. Comparative studies nevertheless demonstrate that physiological deterioration and transport-related adverse events can occur, with reported rates varying according to patient selection, transport setting, and outcome definitions [5,10,13,14,15]. The observed rate in the present cohort may additionally have been influenced by selective or incomplete routine documentation, a recognized limitation of interhospital transport research [16,17,18].

### 4.2. Instability During Transport

Unstable events in this study were frequent: 162/320 (50.6%) patients meeting the definition of serious impairment of physiology experienced transport instability, accounting for 89% of all records with instability. Catecholamine dependency was associated with instability, consistent with prior literature identifying severe circulatory or respiratory impairment as markers of higher risk [19,20,21]. In patients with refractory hypoxemia, pre-transfer stabilization and specialized retrieval, including consideration of veno-venous extracorporeal membrane oxygenation (VV-ECMO), may be appropriate; however, these strategies were not evaluated in this study.

### 4.3. Transfer Durations

Transfer durations increased with clinical complexity, consistent with previous reports [22,23,24].

### 4.4. Strategic Transfers

Strategic and capacity-driven transfers were an important component of the pandemic response. Previous studies and national frameworks suggest that careful patient selection and coordination enable safe strategic transfers, although criteria vary across settings [6,12,25,26,27]. In the present cohort, the routine transport protocol did not consistently record the indication for transfer; however, the supplementary questionnaire identified capacity or resource limitations as a common reason for transfer (n = 187), consistent with shortages of staffed intensive care unit (ICU) and ventilator beds during pandemic peaks. By contrast, only two transfers were explicitly documented as occurring within a cloverleaf region and three as nationwide transfers, indicating that the available documentation does not allow strategic transfers to be comprehensively identified on the basis of these categories. The study population should therefore be understood primarily as isolation-requiring, frequently ventilated patients with COVID-19 transferred in a setting of constrained intensive care resources, rather than as a cohort selected specifically for maximal physiological severity or ECMO support. Because transfer indication and direction were incompletely documented, the present data do not permit a reliable comparison between capacity-driven transfers, transfers for clinical escalation, and step-down transfers. In patients transferred because of worsening clinical status or a need for escalation of care, subsequent physiological deterioration may have reflected the underlying clinical trajectory rather than an effect of the transport itself.

### 4.5. Study Limitations

Several limitations must be considered. First, participation was voluntary and limited to selected providers, introducing potential selection bias, as described in previous observational transport research [17,18].

Second, documentation was heterogeneous and incomplete, introducing potential reporting bias. Inconsistent documentation is a known limitation in interhospital transport studies [16,17,18]. Because undocumented binary predictors were pragmatically coded as absent, incomplete documentation may have resulted in exposure misclassification. Missingness may also have differed between documentation systems and providers; this was not formally assessed, and no sensitivity analysis restricted to standardized DIVI forms was performed. Because documentation format was largely provider-specific, such an analysis would also have been confounded by differences between EMS providers. In addition, the retrospective documentation did not permit a sufficiently reliable and consistent classification of respiratory support across all records to support robust subgroup analyses according to ventilation status. Whether the observed associations apply similarly to invasively ventilated, non-invasively supported, and spontaneously breathing patients therefore remains to be determined in prospectively standardized cohorts. In addition, the composite definitions of transport instability and deterioration were study-specific and have not been externally validated, which limits direct comparison with other transport cohorts.

Third, the use of different documentation formats (DIVI vs. alternative records) has affected comparability, reflecting the lack of standardized national reporting despite prior recommendations [11,17].

Fourth, no data on helicopter transports were available, and the number of participating centers was limited, restricting generalizability [3,21].

Finally, outcome assessment was limited to immediate post-transport changes, and the retrospective design precludes causal inference [6,7].

### 4.6. Practical Recommendations

Based on these results, several practical recommendations can be considered; recommendations extending beyond the observed associations should be interpreted as expert-informed proposals rather than effects demonstrated by this retrospective study. First, interhospital transfers of critically ill patients require structured preparation, appropriate monitoring, and personnel experienced in critical care transport, consistent with previous recommendations for the transfer of critically ill patients [17,18]. Second, standardized, preferably digital, transport documentation should be promoted to improve completeness, comparability, quality assurance, and future research; the need for standardized documentation has been emphasized previously [11,16,17,18]. Third, the observed association between serious impairment of physiology and transport instability, together with the strong association between instability during transport and subsequent deterioration at handover, supports heightened preparation and monitoring of patients with marked physiological impairment [19,20,21]. Fourth, supra-regional coordination and structured patient-selection processes should be further developed for future capacity-driven transfers, in line with previous national and international transfer frameworks [6,12,25,26,27].

## 5. Conclusions

In this multicentre cohort of interhospital intensive care transports from selected German providers, clinically relevant physiological changes during transport and deterioration at handover were not uncommon. The observed associations may help identify patients requiring heightened preparation and monitoring, but the retrospective design does not permit attribution of deterioration to transport itself or conclusions regarding overall transport safety. Heterogeneous documentation, voluntary provider participation, regional clustering, and the absence of helicopter transports limit generalizability. Standardized transport documentation and prospective studies are needed to improve risk assessment and evaluate strategies for future interhospital critical care transfers.

## Figures and Tables

**Table 1:** Multiple linear regression analysis with takeover time (minutes) as dependent variable; all records with takeover time available were retained (n = 360).

	Regression Coefficient (Minutes)	95% CI	*p*-Value
intercept	31.8	22.4–41.1	<0.001
physician seniority	−3.9	−9.1–1.4	0.147
high priority	−9.9	−19.0–−0.9	0.032
catecholamine therapy	9.4	3.6–15.2	0.002
unconsciousness/sedation	7.5	0.3–14.6	0.041
ECMO	21.2	11.6–30.9	<0.001
obesity	14.2	5.1–23.4	0.002
diabetes	7.5	−1.2–16.3	0.092
ventilation	7.8	−2.6–18.2	0.14
serious impairment of physiology	3.8	−1.9–9.4	0.192

**Table 2:** Multiple linear regression analysis with handover time (minutes) as dependent variable; all records with handover time available were retained; instability during transport was added as additional predictor (n = 305).

	Regression Coefficient (Minutes)	95% CI	*p*-Value
intercept	47.8	31.5–64.0	<0.001
physician seniority	5.6	−3.2–14.4	0.211
high priority	−1.3	−16.0–13.4	0.859
catecholamine therapy	10.6	0.5–20.7	0.039
unconsciousness/sedation	−6.8	−19.0–5.5	0.279
ECMO	19.8	3.4–36.1	0.018
obesity	12.3	−3.0–27.5	0.115
diabetes	−0.8	−15.3–13.7	0.915
ventilation	13.7	−4.6–32.0	0.141
instability during transport	−5.1	−13.5–3.2	0.227

**Table 3:** Multivariable logistic regression analysis with instability during transport (n = 66) as dependent variable. Odds ratios are adjusted for the other predictors included in the multivariable model.

	OR	95% CI	*p*-Value
serious impairment of physiology	3.9	1.3–11.7	0.015
analgesics	2.4	1.0–5.7	0.049
crystalloids/colloids	1.8	1.0–3.1	0.047
obesity	1.3	0.5–3.3	0.55
intubated	0.7	0.3–1.4	0.68

**Table 4:** Multivariable logistic regression analysis with deterioration after transport (n = 68) as dependent variable. The independent predictors were the same as in Table 3, with long transport time and instability during transport includes as additional predictors. Odds ratios are adjusted for the other predictors included in the multivariable model.

	OR	95% CI	*p*-Value
serious impairment of physiology	3.0	1.0–8.5	0.042
analgesics	1.3	0.6–3.1	0.49
crystalloids/colloids	1.3	0.7–2.3	0.42
obesity	2.3	0.9–5.6	0.065
intubated	0.7	0.3–1.4	0.31
instability during transport	6.0	3.3–11.2	<0.001
long transport time	1.4	0.3–5.5	0.66

## Data Availability

The data are not publicly available because the original study records exist only as paper-based documentation held by the corresponding author and cannot be shared under the applicable institutional and data governance regulations.

## Supplementary Materials

The following supporting information can be downloaded at https://www.mdpi.com/article/10.3390/jcm15176747/s1, File S1: English translation of the German DIVI intensive care transport report (Version 1.1) used for data collection.

## Abbreviations

The following abbreviations are used in this manuscript:

ARDS	Acute Respiratory Distress Syndrome
ASB	Assisted Spontaneous Breathing
AUROC	Area Under the Receiver Operating Characteristic Curve
BiPAP	Bilevel Positive Airway Pressure
CI	Confidence Interval
CMV	Controlled Mechanical Ventilation
COVID-19	Coronavirus Disease 2019
CPAP	Continuous Positive Airway Pressure
DIVI	German Interdisciplinary Association for Intensive and Emergency Medicine (Deutsche Interdisziplinäre Vereinigung für Intensiv-und Notfallmedizin)
ECMO	Extracorporeal Membrane Oxygenation
FiO_2_	Fraction of Inspired Oxygen
GCS	Glasgow Coma Scale
ICU	Intensive Care Unit
ITW	Intensive Care Transport Vehicle (Intensivtransportwagen)
MAP	Mean Arterial Pressure
OR	Odds Ratio
PaO_2_	Partial Pressure of Arterial Oxygen
PCV	Pressure-Controlled Ventilation
PEEP	Positive End-Expiratory Pressure
P/F ratio	Ratio of Arterial Oxygen Partial Pressure to Fraction of Inspired Oxygen (PaO_2_/FiO_2_)
SARS-CoV-2	Severe Acute Respiratory Syndrome Coronavirus 2
SpO_2_	Peripheral Oxygen Saturation
SPSS	Statistical Package for the Social Sciences
VV-ECMO	Veno-Venous Extracorporeal Membrane Oxygenation
