# Supplementary material for: Factors Associated with Transport Instability and Outcome Deterioration During Interhospital Intensive Care Transfers of Patients with COVID-19 in Germany
Source: J Clin Med. 2026 Aug 31;15(17):6747. doi: 10.3390/jcm15176747 (PMC13566136; doi:10.3390/jcm15176747)
Supplement: Supplementary file 1 [file jcm-15-06747-s001.zip › jcm-4493324-supplementary.pdf]

INTENSIVE CARE TRANSPORT PROTOCOL (DIVI Version 1.1)

Adapted from the German DIVI Intensive Care Transport Protocol – Functional English Translation

MISSION NUMBER | | | | | | | | | |

Site

Date of Operation D D M M Y Y

Health Insurance / Cost Carrier — Date of birth

Patient´s Name / First Name ☐ M ☐ F

Address

ZIP — City

Insurance No. — Patient ID — Status

Establishment No. — Physician No.

☐ Intensive Care ☐ Team Transport ☐ Equipment Transport ☐ Organ Transport

Means of transport — Type

☐ Mobile ICU helicopter ☐ Mobile ICU ☐ ALS ambulance ☐ BLS ambulance (+ emergency physician) ☐ rescue helicopter ☐ physician-staffed ALS ambulance ☐ airliner or commercial plane ☐ ambulance jet

1. Operational Data

Team of physicians

Specialisation ☐ Anaesthesia ☐ Paediatrics ☐ Surgery ☐ Other specialization ☐ Internal medicine

Qualification ☐ Specialist ☐ Physician in further training

Assistant staff 1

Qualification ☐ Paramedic ☐ Nursing staff

Assistant staff 2

Qualification ☐ Paramedic ☐ Nursing staff

Mission assignment

departure/take-off station

arrival at patient's location

start of transport

handover at destination hospital

end of mission

2. Physician-to-Physician Communication

Name of requesting physician — Phone — Fax — Requesting Facility — Ward

name of receiving physician — Phone — Fax — Requesting Facility — Ward

Reason for transfer

from		to
☐	Diagnostics	☐
☐	Intensive care	☐
☐	Surgery/ICU	☐
☐	other reason	☐

originating hospital		receiving hospital
☐	Emergency room	☐
☐	Operating theatre	☐
☐	ICU	☐
☐	General ward	☐
☐	Basic/standard care	☐
☐	Specialist care	☐
☐	Maximum care	☐
☐	Rehabilitation clinic	☐
☐	Transfer/intermediate transport	☐
☐	other	☐

Priority

urgent transport

☐ within < 30 minutes (immediate)

☐ < 2 hours (urgent)

transport scheduled

☐ within < 24 hours (same day)

☐ >24 hours (next day/following days)

Patient Category ☐ High-risk patient ☐ ICU patient ☐ no threat to life

Medical discussion prior to transfer ☐ did not take place

3. Patient Status Before Transport

Neurology ☐ normal

Pupillary function

	right	left
narrow	☐	☐
medium	☐	☐
wide	☐	☐
anisocoria	☐	☐
positive light reaction	☐	☐

Neurological Status

☐ conscious ☐ confused ☐ unconscious ☐ (analgesic) sedated

Glasgow Coma Scale

GCS total score

Paralysis ☐ Yes ☐ No

Meningism ☐ Yes ☐ No

Pain ☐ None ☐ Mild ☐ Severe ☐ Cannot be assessed

Circulation

Blood pressure ___ / ___ mmHg — Heart rate ___ /min

Circulation ☐ Stable ☐ Unstable ☐ Catecholamine dependent

ECG

☐ SR/PM ☐ Supraventr. ES/AV-Bl. II/VES mono

☐ QRR-tachyk./VESpoly/VHF/AV-Bl. III ☐ VT/VF/EMD

Scores ☐ Collected ☐ Adopted

NACA

	no functional limitation	functional limitation conventional therapy	functional limitation compensated maximum therapy	functional limitation decompensated maximum therapy	functional limitation infaust
Central nervous system	☐	☐	☐	☐	☐
Heart	☐	☐	☐	☐	☐
Blood vessels	☐	☐	☐	☐	☐
Lungs	☐	☐	☐	☐	☐
Blood/clotting	☐	☐	☐	☐	☐
Liver	☐	☐	☐	☐	☐
Kidneys/urinary tract	☐	☐	☐	☐	☐

SAPS II

TISS 28

Respiratory status

☐ Cyanosis ☐ Dyspnea ☐ Stridor/spasticity ☐ Craquels ☐

☐ Spontaneous ☐ CMV ☐ PRV ☐ ASB ☐ Inverted

☐ PCV ☐ BIPAP ☐ CPAP ☐ PEEP > 8

other type of ventilation

Oxygen nasal cannula ___ l/min

SaO_2 ___ %

$etCO_2$ ___ mmHg

Ventilation prior to transport

RR

MV ___ l/min

FiO_2

I:E

PEEP ___ cm H_2O

PIP ___ cm H_2O

Pressure support ___ cm H_2O

Blood gas analysis

pO_2 ___ mmHg — pH

pCO_2 ___ mmHg — S-Bic ___ mmol/l — Temp. ___ °C

Comments / special features / laboratory

4. Diagnoses

Primary diagnosis (ICD-10)

Secondary diagnosis (ICD-10)

surgery / intervention (ICPM)

Documents sent with patient: CT/X-ray, findings, valuables

5. Event record

Event

Pulse • — Blood pressure — Defibrillator — Intubation — Chest compressions — Transport T

Monitoring

Heart rate / Blood pressure: 200, 160, 120, 80, 40

RR / MV

PIP / PEEP

I:E

O_2 / FiO_2

SaO_2 / $etCO_2$

Fluid admission: Medication; infusion/transfusion

Fluid output: Urine; Drains

Comments

6. Measures and Equipment

Measures

Cardiovascular

existing ☐ new ☐ Peripheral IV — Number — Location

existing ☐ new ☐ Central Venous Catheter — Number — Location

existing ☐ new ☐ Arterial Line — Location

existing ☐ new ☐ Pulmonary Artery Catheter — Location

existing / new: ☐ ☐ Pacemaker ☐ ☐ CPR ☐ ☐ 12-lead ECG

Respiration (existing / new)

☐ ☐ Oxygen therapy

☐ ☐ Intubation ☐ Oral ☐ Nasal ☐ Tracheostomy

Size

Tube depth/fixation

☐ ☐ Suctioning

Other (existing / new)

☐ ☐ Nasogastric Tube

☐ ☐ Chest Drain ☐ right ☐ left

Size

Position

☐ ☐ Urinary Catheter

Monitoring (existing / new)

☐ ☐ ECG ☐ ☐ Non-invasive BP ☐ ☐ Invasive BP ☐ ☐ Pulse oximetry ☐ ☐ CVP ☐ ☐ PAP ☐ ☐ Capnometry ☐ ☐ ICP ☐ ☐ Temperature ☐ ☐ Blood gas analysis

Equipment used (existing / new)

☐ ☐ Stiffneck ☐ ☐ Vacuum mattress ☐ ☐ Syringe pump ☐ ☐ IABP ☐ ☐ Emergency ventilator ☐ ☐ Intensive ventilator ☐ ☐ ECLA/NO ☐ ☐ Incubator

Medications (existing / new)

☐ ☐ Analgesics ☐ ☐ Antiarrhythmics ☐ ☐ Antiemetics ☐ ☐ Antiepileptics ☐ ☐ Antihypertensives ☐ ☐ Anticoagulants ☐ ☐ Bronchodilators ☐ ☐ Diuretics ☐ ☐ Glucose

☐ ☐ Catecholamines ☐ ☐ Corticosteroids ☐ ☐ Muscle relaxants ☐ ☐ Anesthetics ☐ ☐ Buffers ☐ ☐ Sedatives ☐ ☐ Vasodilators ☐ ☐ Thrombolytics ☐ ☐ Others

Infusions (existing / new)

☐ ☐ Blood products ☐ ☐ Crystalloids ☐ ☐ Colloids ☐ ☐ Others

7. Handover Status After Transport

Neurology ☐ normal

Pupillary function

	right	left
Narrow	☐	☐
Medium	☐	☐
Wide	☐	☐
Unrounded	☐	☐
Positive light reaction	☐	☐

Neurological Status

☐ Conscious ☐ Confused ☐ Unconscious ☐ (Analgesic) Sedated

Glasgow Coma Scale

GCS Total Score

Paralysis ☐ Yes ☐ No

Meningism ☐ Yes ☐ No

Pain ☐ None ☐ Mild ☐ Severe ☐ Cannot be assessed

Circulation

Blood pressure ___ / ___ mmHg — Heart rate ___ /min

Circulation ☐ Stable ☐ Unstable ☐ Catecholamine dependent

ECG

☐ SR/PM ☐ supraventr. ES/AV-Bl. II/VES mono

☐ QRR-tachyk./VESpoly/VHF/AV-Bl. III ☐ VT/VF/EMD

Comments

Respiratory Status

☐ Cyanosis ☐ Dyspnea ☐ Stridor/Spasticity ☐ Craquels ☐

☐ Spontaneous ☐ CMV ☐ PRV ☐ ASB ☐ Inverted

☐ PCV ☐ BIPAP ☐ CPAP ☐ PEEP>8

other type of ventilation

Oxygen nasal cannula ___ l/min

SaO_2 ___ %

$etCO_2$ ___ mmHg

Ventilation prior to transport

RR ___ /min

MV ___ l/min

FiO_2

I:E

PEEP ___ cm H_2O

PIP ___ cm H_2O

Pressure support ___ cm H_2O

Blood gas analysis

pO_2 ___ mmHg — pH

pCO_2 ___ mmHg — S-Bic ___ mmol/l — Temp. ___ °C

8. Outcome

Transportation medically indicated

☐ Yes ☐ No

Description of mission

☐ Transport to hospital

☐ Transfer to/from other emergency services

☐ False alarm

☐ Patient not transportable

☐ Death during transport

Incident coding (AVB): Type — Relevance — Time

Receiving ward/Doctor

Phone/Fax

Signature stamp of the doctor
